# Fibroblast growth factor 10 upregulates the expression of mucins in rat conjunctival epithelial cells

**Published:** 2011-10-26

**Authors:** Mingming Ma, Zhengwei Zhang, Weiran Niu, Wenjing Zheng, Jiang Kelimu, Bilian Ke

**Affiliations:** Department of Ophthalmology, Shanghai Key Laboratory of Fundus Diseases, First People’s Hospital, Shanghai Jiao Tong University, Shanghai, China

## Abstract

**Purpose:**

This in vitro study aimed to gain insight into the function of fibroblast growth factor 10 (FGF10) on the ocular surface, especially its effect on mRNA expression of the mucins *Muc1*, *Muc4*, and *Muc5ac*, and mucin protein synthesis.

**Methods:**

We isolated primary cultured rat conjunctival epithelial cells (Cj-ECs) and treated them with FGF10 (1 ng/ml, 10 ng/ml, 100 ng/ml, and 200 ng/ml) and basic fibroblast growth factor 2 (FGF2; 10 ng/ml) for 24 h or 48 h. The proliferation of Cj-ECs was evaluated by 3-(4,5-dimethylthiazol-2-yl)-5-(3-carboxymethoxyphenyl)-2-(4-sulfophenyl)-2H-tetrazolium (MTS). mRNA levels of *Muc1*, *Muc4*, and *Muc5ac* were determined by real-time PCR. Synthesis levels of MUC1 and MUC4 were measured by western blot. Flow cytometry and Annexin V/PI double staining revealed degrees of apoptosis.

**Results:**

In primary culture, the epithelial cells were compact and cobblestone pavement in shape. Most of the cells were positive for cytokeratin (CK). FGF10 and FGF2 significantly stimulated *Muc1*, *Muc4*, and *Muc5ac* mRNA expression, cell proliferation, and synthesis of MUC1 and MUC4 proteins. FGF10 was more potent than FGF2 in these regards. FGF10 did not restrain the apoptosis of Cj-ECs.

**Conclusions:**

The results of this study demonstrated that FGF10 is associated with the promotion of Cj-EC proliferation and mucin production. The effects of FGF10 on Cj-ECs support a rationale to investigate its therapeutic potential for ocular surface diseases.

## Introduction

Mucins are important structural and functional components of the tear film. The hydrophilic nature of these large and highly glycosylated glycoproteins enable them to play a critical role in the protection of the corneal and conjunctival epithelium [1,2]. Mucins also lubricate the ocular surface during blinking, help to create a smooth surface, and provide a barrier to pathogen infection. Membrane-associated mucins such as MUC1 and MUC4 are expressed in apical stratified epithelium [3,4], while MUC5AC is the most prevalent mucin secreted by goblet cells. MUC5AC especially helps maintain a wet ocular surface that protects against infectious pathogens, and chemical and mechanical trauma [5-8].

Although mucins are important in the maintenance of a healthy ocular surface, abnormal expression of conjunctival mucin has been implicated in disorders such as corneal injury, chronic inflammation, Stevens-Johnson syndrome, dry eye disease and hypovitaminosis A [9,10]. In dry eye patients with Sjögren's syndrome, a significant decrease in the expression of *MUC5AC* mRNA and MUC5AC protein has been reported, suggesting that MUC5AC is important in preventing drying diseases of the ocular surface [9,11]. Furthermore, previous studies performed with animal models suggest that the neuropeptides, cytokines, and growth factors released by nerve termini near the goblet cells promote the proliferation of conjunctival epithelial cells and stimulate mucin production and secretion [12-15].

Members of the fibroblast growth factor (FGF) family are involved in a wide variety of physiologic and pathological processes, including inflammation, repair, and regeneration. FGF2, also known as basic FGF, stimulates cellular proliferation and modulates endothelial cell migration. In addition, FGF2 promotes the recruitment of inflammatory cells to the wound site during wound recovery [16]. FGF10, (or keratinocyte growth factor 2), promotes the growth, proliferation, and differentiation of epithelial cells [17-20], accelerates wound repair [21], regulates organ morphogenesis [22,23], and induces angiogenesis [24]. The carcinogenesis of some organs is closely associated with the abnormal expression of FGF10 [25,26].

FGF receptor 2b (FGFR2b), expressed by epithelial cells, is activated by FGF10 [27]. Binding specificity between FGF10 and the receptor FGFR2b is crucial for the proper regulation of FGF10 functions and a disruption may induce developmental malformations in the lacrimal gland, lung, tooth, heart, limb, thymus, and bone. It has been shown that the embryonic development of the lacrimal and salivary glands in humans is sensitive to FGF10 levels, which are a reflection of the genotype. Patients with autosomal dominant aplasia of the lacrimal and salivary glands (ALSG) present missing or hypoplastic parotid and submandibular glands [28]. The gene for ALSG was mapped to 5p13.2–5q13.1, which coincides with the *FGF10* gene [28]. Furthermore, all patients with ALSG were heterozygous for *FGF10*, and *Fgf10*-heterozygous mouse embryos had no parotid gland, and smaller submandibular glands [29]. FGF10 is also necessary for the morphogenesis of mammary glands and the distal colon [30,31], indicating the crucial importance of *FGF10* in the development of certain organs.

Some studies have shown that corneal injury and inflammation induce the upregulation of *FGFR2b* and *FGF10* [32-34]. In experimental animal models and researches, corneal damage and inflammation was related to an increase of mucin production by the conjunctiva [35,36]. Thus we hypothesized that *FGF10* may play a role in corneal wound healing and mucin synthesis. To gain insight into the function of *FGF10* in the ocular surface, in the present study we evaluated the effect of FGF10 on mRNA expression of *Muc1*, *Muc4*, and *Muc5ac*, as well as on MUC protein synthesis, cell proliferation, and apoptosis, using primary cultured rat conjunctival epithelial cells (Cj-ECs) as an experimental model.

## Methods

### Reagents

RPMI-1640 culture medium, penicillin-streptomycin, Hanks’ balanced salt solution, L-glutamine, trypsin-EDTA, fetal bovine serum (FBS) and TriZol were purchased from Invitrogen (Grand Island, NY). FGF10 was a gift from Xiaojie Wang at the Wenzhou Eye Research Institute (Wenzhou, Zhejiang, China). FGF2 was obtained from R & D Systems (Minneapolis, MN). The Cell Proliferation Assay (MTS) kit came from Promega (Madison, WI). Reverse Transcription and RT–PCR System were purchased from Roche (Mannheim, Germany) and Takara (Otsu, Japan), respectively. All other reagents were obtained from Sigma (St. Louis, MO).

Mouse monoclonal antibody against pan-keratin for immunocytochemistry was from Lab Vision & Neomarkers (Fremont, CA). For western blot analysis, rabbit monoclonal antibody against the rat MUC1 receptor was from Epitomics (Burlingame, CA), monoclonal antibody against rat MUC4 was from Invitrogen (Camarillo, CA), and antibody to rat β-actin (Actb) was from Santa Cruz Biotechnology (Santa Cruz, CA). The secondary antibodies for immunocytochemistry were from Genentech (South San Francisco, CA), and for western blot from Santa Cruz Biotechnology.

### Animals

Male Sprague-Dawley rats (250–300 g) were used in this study and were obtained from Shanghai Laboratory Animal Center (Shanghai, China). Male rats were chosen to avoid possible gender-related differences. Rats were anesthetized with CO_2_ for 1 min, decapitated, and the nictitating membranes and fornix of the conjunctival tissue were surgically removed from both eyes.

The experimental protocols used in this study followed guidelines established by the ARVO Statement for the Use of Animals in Ophthalmic and Vision Research and were approved by the Schepens Eye Research Institute Animal Care and Use Committee.

### Primary culture of rat conjunctival epithelial cells

The removed conjunctival tissues were immediately placed into phosphate buffered saline (PBS) containing penicillin-streptomycin (100 ug/ml), washed 3 times, and finely minced into approximately one cubic millimeter pieces that could be anchored onto culture dishes. Each culture dish contained just enough medium to cover the bottom of the dish, so that the tissues would receive nutrients through surface tension but not float away. The medium consisted of RPMI-1640 medium supplemented with 2 mM L-glutamine, 10% heat-inactivated FBS and 100 ug/ml penicillin-streptomycin. Epidermal growth factor (10 ng/ml) was added on the first day, and 3 days after plating. The medium was changed every 2 days and the tissues were grown under routine culture conditions of 95% O_2_/5% CO_2_ at 37 °C.

Cells were permitted to grow from the tissue plug until spaced nodules were evident, forming a circular pattern around the tissue plug, which was then removed. Meanwhile, all cells that grew outside the circular perimeter were removed by scraping the bottom of the dish with a rubber policeman. Cells were passaged after being trypsinized with 0.05% trypsin-0.53 nM EDTA (pH 7.4). The inoculation density of passaged cultures was approximately 3–5×10^4^ cells per well. Finally, cells were identified as Cj-ECs by the following characteristics: (1) morphology, visualized by inverted phase contrast microscopy; (2) positive staining of pan-keratin by immunocytochemistry; and (3) positive staining by Alcian blue-peroidic acid Schiff (AB-PAS). All experiments used second-passage epithelial cells.

Exogenous FGF10 at concentrations of 1, 10, 100, or 200 ng/ml were added to the culture medium (modified to a low FBS concentration of 1%).

### Immunocytochemistry

Cj-ECs were grown on glass coverslips in 12-well plates. After reaching confluence, the cells were rinsed in PBS, fixed in 100% methanol for 15 min at room temperature, and washed with PBS. The coverslips were incubated in blocking buffer that contained 1% BSA (BSA) and 0.2% Triton-X in PBS for 30 min at room temperature, then incubated with the pan-keratin antibody (1:200 in PBS) for 2 h at 37 °C, then HRP-conjugated second antibody (1:500 in PBS) for 45 min at room temperature. Finally the coverslips were washed three times in PBS and exposed to diaminobenzidene (DAB), then mounted and observed under an inverted phase-contrast microscope (IX71; Olympus, Tokyo, Japan) equipped with a digital camera (Axioplan 2 Imaging, Carl Zeiss, Göttingen, Germany). For the negative control, PBS was substituted for the primary antibody.

### Histochemistry

Neutral and acidic glycoconjugates, which are well established markers of goblet cells, were also present in cultured goblet cells. Cells were grown on glass coverslips in 12-well plates, fixed with 4% paraformaldehyde, and subjected to histochemical analysis using the mucin stain AB/PAS.

### RNA isolation and RT–PCR

Total RNA was isolated from cultured epithelial cells with TRIzol according to the manufacturer’s protocol. One microgram total RNA was used for cDNA (cDNA) synthesis using the Reverse Transcription System (Takara Bio, Otsu, Japan) according to the manufacturer’s instructions. The cDNA was amplified by PCR using primers specific for rat *Muc1*, *Muc4*, *Muc5ac*, and *Actb* in a thermal cycler (PCR Sprint; Thermo Hybaid, Franklin, MA). The sequences of the primers were as follows: *Muc1* sense 5′-ACC ACG GCT ACG TCA GCT ATC AC-3′ and antisense 5′-AGA TGG GCT GCT GAC TTG GAA-3′; *Muc4* sense 5′-GCT TGG ACA TTT GGT GAT CC-3′ and antisense 5′-GCC CGT TGA AGG TGT ATT TG-3′; *Muc5ac* sense 5′-ACC ACC TCC ATC TTG CTG TCA CTC A-3′ and antisense 5′-CCC AGG ATG CCT TTC GTG TTG TCA-3′; *Actb* sense 5′-AAG TTT CAG CAC ATC CTG CGA GTA-3′ and antisense 5′-TTG GTG AGG TCA ATG TCT GCT TTC-3′. *Actb* served as the internal control. Each PCR reaction contained 0.5 uM primers, 200 uM dNTPs, 1.5 uM MgCl_2_, 1.25 U of Taq polymerase, and one microliter cDNA. The parameters were as follows: 5 min at 94 °C, followed by 35 cycles of denaturation for 30 s at 94 °C, amplification for 1 min at the indicated temperature, and extension for 1 min at 72 °C.

### Western blot analysis

Cells (1×10^6^) from primary culture were scraped and homogenized in RIPA buffer (10 mM Tris-HCL pH 7.4, 150 mM NaCl, 1% deoxycholic acid, and 1% Triton X-100) containing protease inhibitors (100 μl/ml phenylmethylsulfonyl fluoride, aprotinin 30 μl/ml and sodium orthovanadate 100 nM). Homogenized cells were sonicated and centrifuged at 2,000× g for 15 min at 4 °C. Proteins in the supernatant were separated by 8 or 12% sodium dodecyl sulfate-PAGE (SDS–PAGE) and transferred onto polyvinylidene fluoride (PVDF) membranes. The membranes were blocked for 2 h at room temperature in 5% nonfat dried milk in buffer containing 10 mM Tris-HCL (pH 8.0), 150 mM NaCl, and 0.05% Tween-20, and then incubated with primary antibody for 2 h at room temperature or overnight at 4 °C at the following dilutions: MUC1 antibody at a dilution of 1:300 in TBST, MUC4 antibody at 1:500, and β-actin antibody at 1:1000. The membranes were washed three times with TBST and incubated with HRP-conjugated secondary antibody at a dilution of 1:5,000 for 2 h. Finally the membranes was washed 3 times with TBST and developed using the enhanced chemiluminescence method.

### Cell proliferation assay

Rat Cj-ECs in primary culture were trypsinized and seeded in 96-well plates at a density of 2×10^4^ cells/well. Cells were grown to approximately 60% confluence and cultured in serum-free RPMI medium for 24 h. Cells were then treated with 1% FBS (the negative control), 10 ng/ml FGF2 (the positive control), or 1, 10, 100, or 200 ng/mL FGF10 and incubated for 24 h or 48 h. Cell proliferation was determined by the 3-(4,5-dimethylthiazol-2-yl)-5- (3-carboxymethoxyphenyl) -2-(4-sulfophenyl)-2H-tetrazolium (MTS) assay, a colorimetric assay for the quantification of cell proliferation and viability. Cells were incubated in MTS (20 ul) for 1 h; the absorbance was then read at 490 nm.

### Apoptosis assay by Annexin V/PI

Cells were harvested (72 h after exposure to FGF10 or FGF2), washed with cold PBS and resuspended in binding buffer (0.01 M HEPES pH 7.4; 0.14 M NaCl; 2.5 mM CaCl_2_) at a density of 1×10^6^/ml. Cells (100 μl) were mixed with 5 μl fluorescein isothiocyanate (FITC)-Annexin V and 10 μl propidium iodide (PI; 20 μg/ml) and incubated for 20 min in the dark at room temperature. Cells (1×10^4^) were analyzed for each sample by flow cytometry (FACS Caliber, Becton Dickinson, Heidelberg, Germany). Cell apoptosis was assayed using Cell Quest software (BD Biosciences, San Jose, CA).

### Data analysis

The amount of mucin proteins were expressed as a ratio to basal conditions, which were set at a value of one. Data were represented as mean±standard deviation (SD) in the text or mean±standard error of the mean (SEM) in figures, and analyzed by Student’s *t*-test. A probability (p)-value <0.05 was considered statistically significant.

## Results

### Characterization of cultured rat epithelial cells

**Figure 1:**
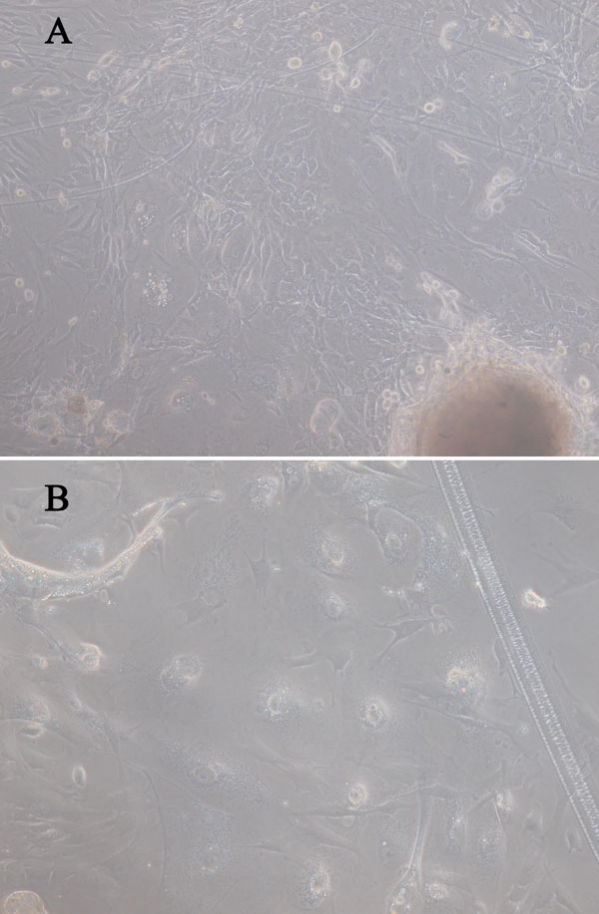
Phase contrast micrographs illustrating the morphology of rat conjunctival explants grown in vitro. Magnification: (**A**) 100×, (**B**) 400×.

After 10 to 14 days of culture, the rat primary Cj-ECs were approximately confluent and their morphology appeared compact and cobblestone pavement in shape (Figure 1).

**Figure 2:**
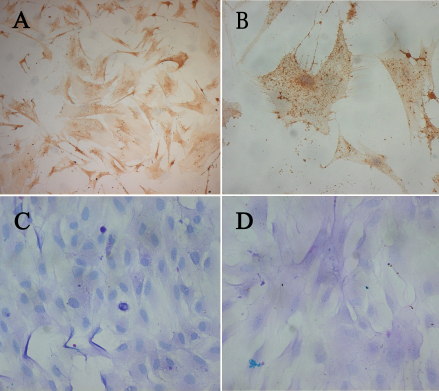
Photomicrographs of rat conjunctival epithelial cells (Cj-ECs) in culture. **A**, **B**: Cj-ECs stained intensely for pan-keratin, a marker of conjunctival epithelial cells. **C**, **D**: Histochemical reactivity of primary culture of Cj-ECs to AB/PAS. Goblet cells stained intensely with AB/PAS, indicating the presence of both neutral (pink) and acidic (blue) glycoconjugates associated with cells. Magnification: (**A**, **C**), 100×; (**B**, **D**), 400×.

Immunocytochemistry staining revealed that almost all the Cj-ECs were positive for pan-keratin (Figure 2A,B), a marker of epithelial cells. In addition, these cells were positive for AB/PAS staining (Figure 2C,D), indicating that they produced neutral and acidic mucins.

### FGF10 stimulated conjunctival epithelial cell proliferation

**Figure 3:**
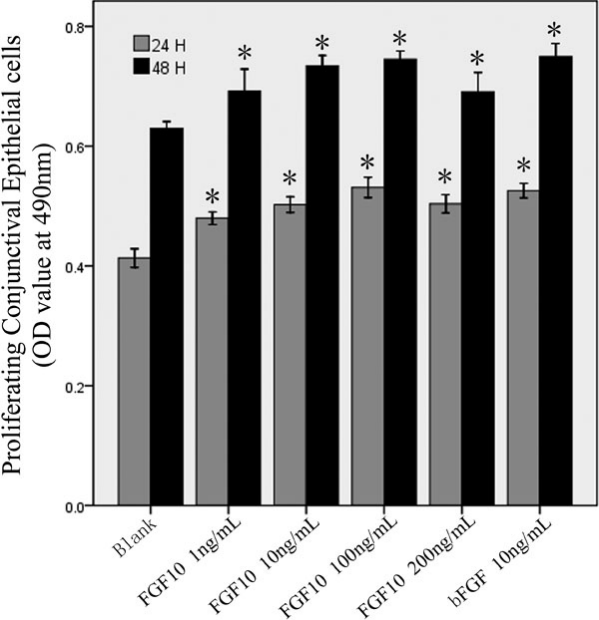
FGF10 stimulates epithelial cell proliferation. Epithelial cells from primary culture were stimulated with FGF10 (1, 10, 100, or 200 ng/ ml) or FGF2 (bFGF; 10 ng/ ml) for 24 h or 48 h. Cell proliferation was measured using MTS. Data were mean±SEM from 6 independent experiments. *p<0.05 versus blank (cells treated with 1% FBS).

The MTS assay demonstrated that FGF10 stimulated the proliferation of Cj-ECs significantly in a dose-dependent manner, compared to the negative control (1% FBS; p<0.05; Figure 3). As the positive control, FGF2 significantly stimulated the proliferation of Cj-ECs, compared to the negative control (24 h and 48 h, p<0.001). There was no significant difference between FGF10 and FGF2 in stimulating cell proliferation (24 h, p=0.538; 48 h, p=0.555).

### FGF10 upregulated the expression of mucin in rat primary conjunctival epithelial cells

**Figure 4:**
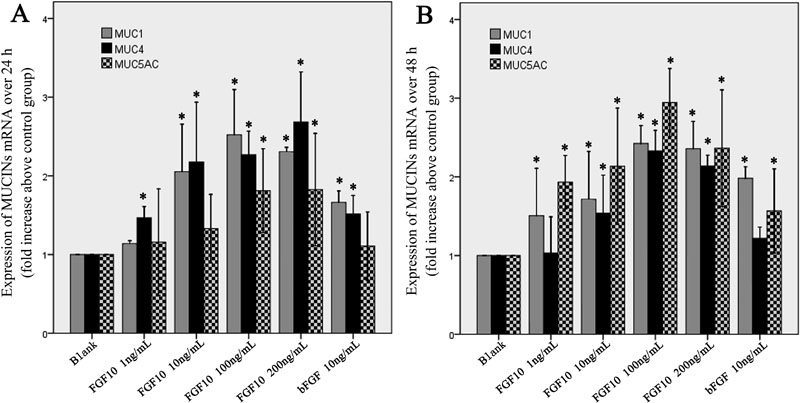
FGF10 increases the expression of *Muc1*, *Muc4*, and *Muc5ac* mRNA. RT–PCR analysis showed that the expressions of *Muc1*, *Muc4*, and *Muc5ac* mRNA were upregulated significantly in epithelial cells treated with FGF10 (1, 10, 100, or 200 ng/ml) for 24 h (**A**) or 48 h (**B**), compared to the negative group. Data were the mean±SEM of 3 experiments performed in quadruplicate. *p<0.05 versus blank (cells treated with 1% FBS).

To investigate the effect of FGF10 on *Muc1*, *Muc4*, and *Muc5ac* mRNA expression, Cj-ECs were treated with FGF10 for 24 h (Figure 4A) or 48 h (Figure 4B). Relative RT–PCR analysis showed that the expressions of *Muc1*, *Muc4*, and *Muc5ac* were upregulated significantly by FGF10, compared to the negative control (p<0.05). In particular, FGF10 at the dose 100 ng/ml was more potent than FGF2 in upregulating the expressions of *Muc1*, *Muc4*, and *Muc5ac* (24 h: *Muc1* p=0.048, *Muc4* p=0.037, *Muc5ac* p=0.046; 48 h: *Muc1* p=0.043, *Muc4* p=0.011, *Muc5ac* p=0.041).

**Figure 5:**
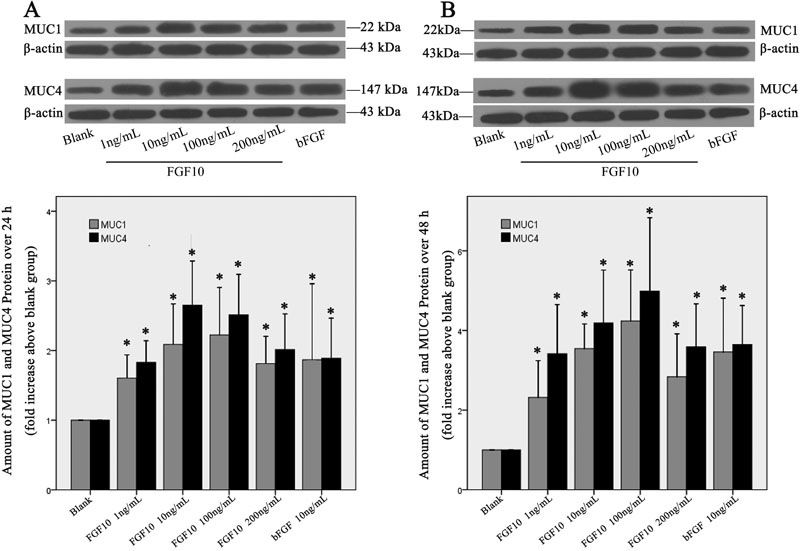
FGF10 stimulates the synthesis of MUC1 and MUC4. Epithelial cells from primary culture were stimulated with FGF10 (1, 10, 100, or 200 ng/ml) or bFGF (10 ng/ml) for 24 h (**A**) or 48 h (**B**). Cells were scraped and analyzed by western blot with antibodies against MUC1 and MUC4. Representative blots are shown in **A** and **B**. Data shown in the chart are mean±SEM from 6 independent experiments. *p<0.05 versus blank (cells treated with 1% FBS).

To confirm these observations, we performed western blot to examine the protein levels of MUC1 and MUC4 in Cj-ECs in conditioned media treated with FGF10 for 24 h (Figure 5A) or 48 h (Figure 5B) and the results showed that the FGF10 treatment led to a significant increase of both MUC1 and MUC4 synthesis compared to the negative control (p<0.05). Furthermore, the 100 ng/ml dose of FGF10 was more potent than FGF2 in stimulating the synthesis of MUC1 and MUC4 (24 h: MUC1 p=0.036, MUC4 p=0.046; 48 h: MUC1 p=0.021, MUC4 p=0.035).

### FGF10 had no obvious effect on apoptosis of rat conjunctival epithelial cells

FGF10-treated cells were stained with Annexin V/PI and gated into LR (Low Right), UR (Upper Right), LL (Lower Left) and UL (Upper Left) quadrants. The cells in the LR and UR quadrants were considered early apoptotic (Annexin^+^/PI^-^) and late apoptotic (Annexin^+^/PI^+^), respectively; altogether they demonstrated apoptosis. The cells in LL and UL quadrants were live and necrotic, respectively.

**Figure 6:**
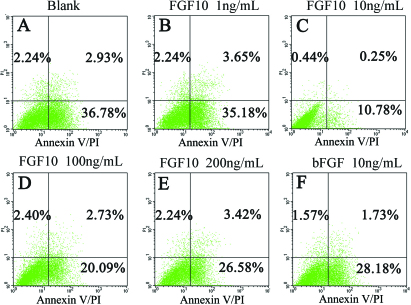
FGF10 has no obvious effect on apoptosis in epithelial conjunctival cells. Primary cultured cells were incubated with FGF10 or bFGF at the indicated concentrations for 72 h. Each sample was stained by Annexin V/PI and analyzed by flow cytometry. The ratio was shown in each quadrant.

The results showed that although the apoptosis rate decreased in FGF10- or FGF2-treated cells compared to the negative control (Figure 6), the difference was not significant (1 ng/ml, p=0.307; 10 ng/ml, p=0.172; 100 ng/ml, p=0.103; 200 ng/ml, p=0.077; bFGF, p=0.295). Thus, FGF10 did not appear to induce any significant changes in apoptosis of rat Cj-ECs.

## Discussion

In this in vitro study we demonstrated that FGF10 promoted the proliferation of rat Cj-ECs and stimulated the expression and synthesis of mucins. These data suggest that conjunctival epithelial cells, which have been shown to express the growth factor receptor FGFR2b [37], respond to FGF10. Although many functions of FGF10 have been documented, the potential role of FGF10 in Cj-ECs remains unexplored [17-23]. Time-dependent and dose-dependent proliferation of Cj-ECs induced by FGF10 was demonstrated in the present study. The effect of FGF10 on the proliferation of Ci-ECs appears to be as dramatic as FGF2, which has been widely used in clinical practice for its ability to stimulate cellular proliferation, cell migration, and wound recovery [16]. Therefore, for the first time we showed that FGF10 acts similarly to FGF2 in cellular proliferation of Cj-ECs.

By real-time PCR and western blot analysis we presented the first evidence that FGF10 stimulates mucin expression and production in rat Cj-ECs. We found that Cj-ECs, treated with FGF10 for 24 or 48 h, expressed high levels of *Muc1*, *Muc4*, and *Muc5ac* mRNA compared with the negative control. In contrast, the mRNA expression of these mucins in FGF2-treated cells was lower than FGF10-treated cells, indicating that FGF10 is more potent than FGF2 in the upregulation of mucins. In addition, by western blot we showed that FGF10 increased the synthesis of MUC1 and MUC4 significantly after 24 and 48 h, compared with the negative control and FGF2.

MUC5AC, produced and released by goblet cells, not only maintains a wet ocular surface, but also stabilizes fluids, and provides a physical and chemical barrier that protects the ocular surface from infectious pathogens, desiccation, and mechanical, chemical, and thermal trauma [2,5]. Further studies are needed to determine whether FGF10 regulates the differentiation of conjunctival epithelial cells into goblet cells. However, FGF10 has an enormous potential in improving the condition of the ocular surface and as an auxiliary treatment in some diseases of the eye.

The regulation of apoptosis is an important component of tissue remodeling. Previous studies have demonstrated that FGF10 attenuated DNA damage and apoptosis of epithelial cells in part by MEK/ERK-dependent signaling that affects the mitochondria-regulated death pathway [38,39]. Therefore, in the present report we investigated apoptosis in Cj-ECs. The Annexin V/PI assay showed that the ratio of apoptotic cells in the group treated with FGF10 for 72 h decreased, but not significantly, compared with the negative control. We conjecture that the effect of FGF10 in promoting proliferation may protect Cj-ECs from apoptosis.

This study had some limitations. Notably, the concentrations of FGF10 used may not be physiologically relevant. Another concern is that epithelial cells grown in vitro may respond differently from cells in vivo. Nevertheless, since there is no similar report yet on the effect of FGF10 in Cj-ECs, our results indicate a potential for FGF10 in treating ocular surface diseases such as dry eye, ocular surface injury, inflammation, and others.

In summary, data reported in this study indicate that FGF10 may exert pleiotropic effects in cultured Cj-ECs: maintaining the survival of Cj-ECs, stimulating conjunctival mucin production, and restraining Cj-EC apoptosis. These biologic functions associated with FGF10 in improving ocular surface conditions suggest that FGF10 is a promising candidate for the treatment of ocular surface diseases.
